# Dr. O. E. Evans

**Published:** 1912-01

**Authors:** 


### OBITUARY

Dr. O. E. Evans, formerly of Akron, died of paralysis in Guthrie, Iowa, November 21, 1911.

